# Val50Ala variant of familial amyloid neuropathy – a rare case in the Czech Republic

**DOI:** 10.1186/1750-1172-10-S1-P24

**Published:** 2015-11-02

**Authors:** Tomas Pika, Pavla Latalova, Helena Hulkova, Patrik Flodr, Vladimir Mejzlik, Vlastimil Scudla

**Affiliations:** 1 Faculty of Medicine and Dentistry, Palacky University Olomouc and Olomouc University Hospital, Department of Hemato-oncology, 77900, Olomouc, Czech Republic; 2 Faculty of Medicine and Dentistry, Palacky University Olomouc and Olomouc University Hospital, Department of Clinical and Molecular Pathology, 77900, Olomouc, Czech Republic; 3 General University Hospital Prague, Institute of Inherited Metabolic Disorders, 12808, Prague, Czech Republic; 4 Centre of Cardiovascular Surgery and Transplantation Brno, Centre of Cardiovascular Surgery and Transplantation Brno, 656 91, Brno, Czech Republic

## Background

Hereditary amyloidosis represents approximately 4% of the total cases of amyloidoses. The most frequent familial type is caused by deposition of mutated transthyretin (TTR, prealbumin). So far it has been identified more than 100 mutations in the transthyretin gene and type of causal mutation is also characterized by a clinical picture of the disease. The most common variant is a neuropathic disease. Characteristic feature is an endemic occurrence with very low incidence in the Central Europe countries.

## Methods

The aim of this communication is to present case of a patient with an inherited form of the TTR amyloidosis which is extremely rare in the Czech Republic.

## Results

25-year-old patient was examined for a history of two years lasting and gradually progressing paresthesias of the lower limbs associated with instability, reduced muscle strength, dysesthesias and peroneal gait. In the last year, also present with intermittent dyspepsia, loss of weight was 10 kg (BMI 16.3, BMI 717.2). On the basis of clinical and EMG finding he was treated as CIDP with i.v. pulses of methylprednisolone, followed by maintenance therapy with prednisone and azathioprin. Afterwards the dyspepsia developed. Gastric endoscopy with biopsy revealed massive deposits of amyloid. Family history clarify amyloidosis with neurological impairment in the patient's mother. Immunohistochemistry confirmed strong positivity of transthyretin in amyloid masses. The sequencing of TTR gene confirmed mutation c.149T˃C with the effect on the coding sequence Val50Ala in heterozygous status. The patient was then registered on the waiting list for OLT. Due to rapid progression of neuropathic involvement despite conservative therapy, therapy with Vyndaqel cps. (Tafamidis meglutime, Pfizer) was initiated at a dose of 20mg per day. Therapy led to a halt of further progression of neuropathic disability, improvement of nutrition and overall improvement of the condition of the patient. 19 months after diagnosis, OLT was performed with good postoperative course. As complications, the early cortico-sensitive rejection episode and bronchopneumonia developed. Currently, the patient is intensively rehabilitating with gradual regression of neuropathic symptoms and improvement of nutritional status (BMI 17.9, mBMI 841).

## Conclusions

Although the Czech Republic is not endemic country for the incidence of familial TTR amyloidosis, it is important to consider this clinical entity, especially in the case of family history. Correct differential diagnosis belongs to the fundamental aspects of care for these patients.

Supported by IGA MZ CR NT 12451/5, NT 14400.

